# Primary Gliosarcoma of the Cerebellum in a Young Pregnant Woman: Management Challenges and Immunohistochemical Features

**DOI:** 10.1155/2019/7105361

**Published:** 2019-07-16

**Authors:** Marco Meloni, Salvatore Serra, Giulia Bellisano, Nikolaos Syrmos, Sanjeeva Jeyaretna, Mario Ganau

**Affiliations:** ^1^Department of Neurosurgery, San Francesco Hospital, Nuoro, Italy; ^2^Department of Pathology, San Francesco Hospital, Nuoro, Italy; ^3^Department of Neurosurgery, Aristotle University of Thessaloniki, Macedonia, Greece; ^4^Nuffield Division of Clinical Neurosciences and Department of Neurosurgery, Oxford University Hospitals, Oxford, UK

## Abstract

**Background:**

Gliosarcoma (GS) represents a rare, high-grade (WHO Grade IV), central nervous system neoplasm, characterized by a very poor prognosis. Similar to other high-grade gliomas, GS affects mainly adults in the 5^th^-7^th^ decade of life and presents a higher incidence in males. The most reported locations of GS are the temporal lobe and the frontal lobe, while only eight cases of GS originating from the posterior cranial fossa are reported in the literature.

**Case Description:**

We report the first case occurring during pregnancy in a 33-year-old patient. Diagnosis was obtained on the 15^th^ week of gestation when patient presented with signs and symptoms of life-threatening raised intracranial pressure. Surgical excision was followed by early recurrence and eventually disease progression because the patient refused adjuvant treatment to save her fetus.

**Conclusions:**

GS should be considered in the differential diagnosis of posterior cranial fossa tumors with radiological features of meningioma or glioblastoma, even in young patients. To this regard, sarcomas, solitary fibrous tumors, and even metastases should be considered, especially in light of the tendency of GS to give rise to extracranial localizations. Whenever an aggressive management with radical excision and adjuvant treatment is not safely achievable, disease progression is likely to be unavoidable.

## 1. Introduction

Gliosarcoma (GS) was firstly described by Stroebe in 1895 as a double face neoplasm, composed, respectively, by glial and mesenchymal elements [1], and subsequently accepted as biphasic tumors after the in-depth histological analysis conducted by Feigin and Gross [2]. The 2016 WHO classification defines GSs as rare, high-grade (Grade IV), central nervous system (CNS) neoplasms, characterized by clearly identifiable gliomatous and metaplastic mesenchymal components [3]. The epidemiological data available in the English literature show that GS, similar to glioblastoma multiforme (GBM), usually affects adults in the 5^th^-7^th^ decade of life with a higher incidence in males [4]. GS may develop de novo, being properly called primary GS, or may result as a transformation of recurrent GBM, showing a remarkable local aggressiveness and a greater propensity for extracranial metastases [5–7]. Recent studies, aimed at identifying highly specific neoplastic biomarkers, hypothesized the monoclonal expansion of a sarcomatous component or the aberrant mesenchymal differentiation of malignant gliomas as the most likely pathophysiology origins of these tumors [8–10]. The most reported presenting locations of primary GS are the temporal lobe and the frontal lobe, while only eight cases of primary GS originating from the posterior cranial fossa are reported in the literature [6, 11–17]. In this report, we describe a case of primary GS in a young pregnant woman: this is the ninth case affecting the posterior cranial fossa and the only one, to our knowledge, affecting a young pregnant woman.

## 2. Case Report

**Figure 1:**
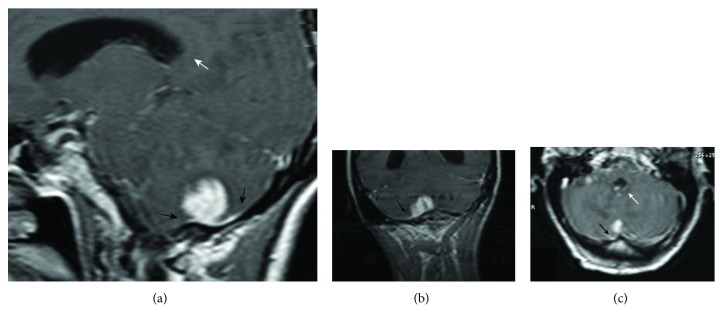
Preoperative T1 postgadolinium injection MRI (a) sagittal, (b) coronal and (c) axial, showing a meningioma-like lesion, localized at the base of the posterior cranial fossa and reaching the cortex of the right cerebellar hemisphere. Note the dural tail (black arrow) and the dilatation of the ventricular system (white arrow).

A 33-year-old pregnant woman (G_2_P_1_A_1_) on the 15^th^ week of gestation was admitted to our Emergency Department with a 3-week history of headache and unsteadiness followed by rapid worsening within 5 days characterized by projectile vomiting, confusion, and psychomotor agitation. A brain magnetic resonance imaging (MRI) scan showed tetraventricular hydrocephalus secondary to obstruction of the cerebrospinal fluid through the foramina of Luschka and Magendie caused by a homogenously enhancing cortical-subcortical lesion localized on the right cerebellar hemisphere, responsible for perilesional edema and characterized by evidence of dural infiltration. The latter, initially mistaken for dural tail, oriented toward the suspicion of posterior fossa meningioma (see Figure 1).

**Figure 2:**
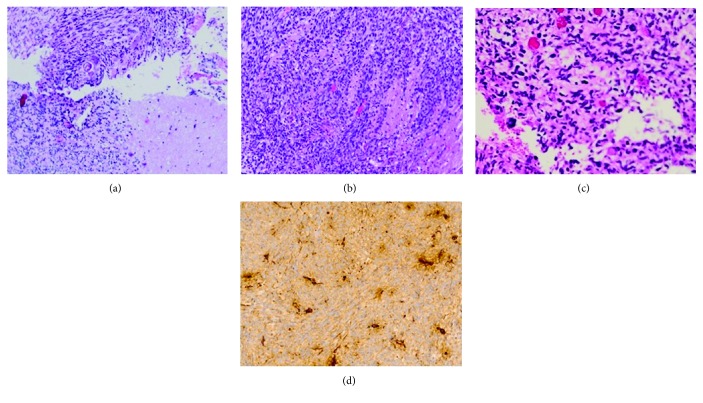
Sections of the tumor showing distinct gliomatous and sarcomatous areas. (a) Cerebellar normal tissue and neoplastic proliferation (H&E magnification ×10); (b) neoplastic spindle elements, consolidated to bundles (H&E magnification ×10); (c) neoplastic elements show cellular and nuclear morphologic atypia with hyperchromic nuclei (H&E magnification ×20); (d) GFAP-positive immunohistochemical reaction (magnification ×40).

Given the critical clinical and radiological scenario, surgical excision was expedited. The patient successfully underwent a suboccipital craniectomy in a sitting position, with the insertion of external ventricular drain (EVD) through the right Keen's point and gross-total removal of the lesion, which resembled an aggressive glioma rather than a meningioma. The general anesthesia was carefully tuned to avoid any impact on the fetus' wellbeing; the postoperative course was uneventful, the EVD was removed within 1 week, and the patient experienced a full recovery with unremarkable neurological status at the time of discharge from the hospital. The histology and the immunohistochemistry analysis surprisingly gave a final diagnosis of GS (see Figure 2); this was confirmed following a second opinion sought from a center of excellence for neuropathology. The consensus from a multidisciplinary team involving gynecologists, neurosurgeons, and oncologists was to have the best interest meeting with the patient and family to decide how to handle the challenges of the adjuvant treatment. Given the patient's decision not to interrupt her pregnancy, no chemotherapy or radiotherapy could be carried out despite the aggressive histology would have warranted them. Following the birth of a healthy baby girl, the patient was transferred to the Radiotherapy Unit to start her first cycle of temozolomide along with conventional radiotherapy. Unfortunately, the almost immediate onset of generalized seizures represented the first flag of disease progression, which led to a sudden and irreversible clinical decline followed by the patient's death within few weeks. She was survived by an inconsolable husband and never managed to see her baby safely discharged from the neonatal intensive care unit.

## 3. Discussion

While several cases of supratentorial primary GS have been reported in literature, only eight cases with a posterior cranial fossa localization have been described so far (see Table 1). This demonstrates how rare the infratentorial localization of this aggressive neoplasia could be. The first case had been described in 1990 on a 62-year-old patient, presenting with ataxia and adiadokinesia due to a cerebellar lesion which was initially mistaken for a malignant fibrous histiocytoma [14]. Following this initial report, between 1993 and 2016, a total of seven additional cases have been described, one of which in a pediatric patient [13]. Interestingly, those lesions have many commonalities: from a clinical perspective, they can either manifest with cerebellar symptoms or more abruptly with signs of raised intracranial pressure; from a pathological perspective, they tend to be locally aggressive, often with dural infiltration, and usually show intralesional necrosis associated with varying degrees of hemorrhage.

On CT scan, GSs tend to appear as slightly hyperdense lesions with perifocal edema and marked homogeneous contrast enhancement, whereas on T1WI and T2WI MRI sequences, they usually show low and high signals, respectively [11, 18]. Overall, from a radiological perspective, their homogeneous enhancement post gadolinium injection and their dural attachment often raise the suspicion of meningiomas, sarcomas, and solitary fibrous tumors including hemangiopericytomas [19–23]. Not surprisingly, the meningioma- or GBM-like macroscopical appearance of GS has been highlighted in several reports [7, 24]. Although the dural tail is actually one of the most frequent radiological features of GS, the sharply demarcated or irregular borders, and the remarkable perilesional edema, should always put metastases among the possible differential diagnosis [6–8]. This is particularly relevant in light of the potential extracranial localizations of GSs described in many cases of supratentorial primary lesions but also in one case from the infratentorial series described in Table 1 [16]. GSs pose several technical challenges to the operating team: they tend to be highly vascularized, carrying a significant risk of intraoperative blood loss and postoperative hematoma [25, 26]. Those considerations pinpoint the complexity of tumor removal and the need to achieve a satisfactory hemostasis before closure, especially in light of the common tumoral infiltration beyond the pseudocapsule [26]. To this regard, the use of visual aids such as the introduction into the surgical cavity of a probe for intraoperative ultrasound or a 30-degree angle endoscope assumes particular relevance [27, 28]. On the other hand, adhering to state-of-the-art prophylactic protocols for prevention of thromboembolism in neurosurgery, and delaying low molecular heparin for 24-48 hours, ensures maximal chances to avoid deep vein thrombosis or pulmonary embolism, as well as postoperative intracranial bleeding [29–31]. The case described here was particularly challenging due to concomitant pregnancy: usually pregnant patients require semisitting, supine, or right lateral position, whereas the prone and left lateral positions are strictly contraindicated due to the gravid uterus pressure on the inferior vena cava, aorta, and iliac vessels during the surgical procedure. In our case, the sitting position was chosen because it could provide optimal approach to the base of the posterior cranial fossa, while guaranteeing a cleaner operating field. The surgical and anesthesiological team took all measures to prevent air embolism including Doppler ultrasonography and strict monitoring of end-tidal carbon dioxide concentration.

The in-depth immunohistochemical analysis conducted in our case expands on the pathological data previously described [32]. Of note, in the present case, we were able to confirm the intralesional coexistence of distinct gliomatous and sarcomatous areas, with diffuse cellular and nuclear morphological atypia, hyperchromic nuclei, high mitotic ratio, and a high Ki67 proliferation index. Those macro- and microscopical features have been widely confirmed in supratentorial GSs; however, our report acquires even more relevance given the lack of a methodological description of the histological and immunohistochemical analysis in the other infratentorial cases described so far. Of note, some authors correlated the prevalence of the sarcomatous component found on histology (>50%) with the firm, well-demarcated appearance of primary GS and similarly to our case highlighted that this component tend to be correlated to a more prominent angiogenesis than the gliomatous one [33]. In our patient, the neoplastic appearance of glial cells and the immunohistochemical positivity for GFAP allowed to rule out fibrosarcoma/malignant fibrous histiocytoma; nonetheless, it is worth mentioning that whenever in doubt an additional reticulin staining could help in identifying the sarcomatous areas, clearly distinguishing the two different components of GS.

The prognosis of GSs is utterly dismal, and our case is a perfect example of how fast this tumor can progress if not adequately treated with adjuvant chemo/radiotherapy after its primary excision. Stereotactic Gamma Knife radiosurgery has been described following subtotal resections or in case of local recurrence despite an initial management with conventional radiotherapy [34–36].

The most noticeable research projects aimed at improving outcomes of GS are currently attempting to focus on new drugs with an antiangiogenesis profile [37–40]. These ongoing efforts pivot on successful strategies already in use for other high-grade gliomas and are capitalizing on the advances of nanotechnology and immunotherapy. Unfortunately, though, the mixed results obtained so far, along with the aggressiveness of this histotype, still make the treatment of GSs one of the greatest neurooncological challenges.

One final note revolves around the correlation between pregnancy and brain tumors: a large retrospective case series of patients with gliomas produced by the French Glioma Study Group suggests that tumor growth accelerates in 80% of patients and grade may evolve during pregnancy, resulting in a significantly higher frequency of seizures which pose specific challenges to the treating team in terms of pharmacological choices [41]. In fact, patients and their partners need to be informed about the possible adverse impact on long-term neurodevelopment of the newborn following in utero exposure to antiepileptic drugs such as sodium valproate. The latest guidelines on the management of epilepsy in pregnancy issued by the Royal College of Obstetricians and Gynaecologists in the United Kingdom states that in utero exposure to carbamazepine and lamotrigine does not appear to adversely affect neurodevelopment of the offspring, while there is very little evidence for levetiracetam and phenytoin [42].

The biologic explanation for tumor growth during pregnancy stems from the observation that multiple hormones and growth factors produced during fetal development also enhance the oncogenesis cascade: for instance, the production of angiogenic factors such as placental growth factor which correlates with gliomas has been widely established [43]. Whereas estrogen and progesterone receptors could be dosed in patients with diagnosis of meningiomas, we found no evidence in the literature for their perioperative dosage in glioma patients [44].

Overall, the open questions regarding the challenges of treating pregnant women with new diagnosis of gliomas revolves around (a) the decision to discourage continuation of pregnancy and when to do so, (b) what monitoring plan is more appropriate for the mother and her fetus, and (c) which chemo- and radiation therapy protocol to suggest after pregnancy. Given the relevance of this clinical scenario, the most recent systematic review concluded that a multicenter individual patient level meta-analysis collecting granular information on clinical management and related outcomes is needed to provide scientific evidence for clinical decision-making in pregnant glioma patients [45].

## 4. Conclusions

Although the posterior cranial fossa remains a rare localization for primary GS, this aggressive tumor should be considered in the differential diagnosis of meningiomas, gliomas, sarcomas, and isolated fibrous tumors, even in young patients. State-of-the-art surgical techniques such as the introduction into the surgical cavity of a probe for intraoperative ultrasound or a 30-degree angle endoscope assume particular relevance to maximize resection and minimize the risk of intraoperative blood loss and postoperative hematoma. While maximal safe resection is fundamental in the management of patients with GS, without adjuvant chemo/radiotherapy the risk of early recurrence/progression of the disease is remarkably high. The case presented here was particularly challenging given the understandable desire of our patient to carry on with her pregnancy. In fact, this situation obliged the patient, as well as each medical practitioner involved in her management, to face a series of ethical questions, where compassionate care had to take priority over clinical pragmatism.

## Figures and Tables

**Table 1:** Clinical characteristics of primary GS cases with a posterior cranial fossa localization reported in the literature.

Case	Age/gender [Ref.]	Clinical presentation	MRI features	Macro-/microscopic anatomy
1	62/M [14]	Ataxia, adiadocokinesia	Not reported	Firm tumor, with dural adhesion to the tentorium cerebelli
2	71/M [11]	Ataxia	Multiple lesions; perifocal edema; homogenous enhancement after gadolinium injection; broad base in contact with the dura mater	Firm, hemispheric well-circumscribed tumor, adherent to the dura; the superficial portion appeared sharply demarcated from the adjacent cerebellar tissue, intralesional hemorrhage/necrosis.
3	80/M [15]	Intracranial hypertension	Solid, homogeneously enhancing mass in the vermis and left cerebellar hemisphere; peritumoral edema causing mass effect and compression on the IV ventricle	Firm and pseudo-encapsulated lesion without attachment to pia or dura; marginal hemorrhage and intralesional necrosis
4	70/F [12]	Intracranial hypertension	Cerebellar intra-axial lesion, with a smooth and slightly lobulated outer layer; minimal peritumoral edema; heterogeneous enhancement after gadolinium injection	Relatively well-circumscribed and firm mass with areas of necrosis and hemorrhage
5	68/M [6]	Not reported	Not reported	Discrete lesion with GBM-like characteristics
6	11/F [13]	Ataxia, intracranial hypertension	Irregularly enhancing lesion located in the cerebellar vermis but characterized by bilateral extension; homogenous enhancement after gadolinium injection	Firm lesions reaching the surface of the cerebellum; white, glistening with areas of hemorrhage and necrosis
7	57/M [16]	Intracranial hypertension	Solid lesion, isointense to the brain parenchyma on T1WI, hyperintense on T2WI, peripheral homogeneous enhancement after gadolinium injection	Firm intra-axial lesion without attachment to the dura mater
8	71/F [17]	Intracranial hypertension	Solid, homogeneously enhancing, hemorrhagic mass in the cerebellopontine cistern	Well-circumscribed mass with intralesional hemorrhage
9	33/F [present case]	Intracranial hypertension	Homogenously enhancing cortical-subcortical lesion localized on the right cerebellar hemisphere, responsible for perilesional edema and characterized by evidence of dural infiltration	Well-circumscribed and firm mass; white, glistening, with intralesional evidence of necrosis
